# The receptor landscape of influenza A viruses

**DOI:** 10.1128/jvi.01443-25

**Published:** 2026-07-16

**Authors:** Kevin Ciminski, Peter Reuther, Rik Rijkers, Robert P. de Vries, Martin Schwemmle

**Affiliations:** 1 Institute of Virology, University Medical Center of Freiburg 221229, Freiburg, Germany; 2 Faculty of Medicine, University Medical Center of Freiburg 74919 , Freiburg, Germany; 3 Department of Chemical Biology & Drug Discovery, Utrecht Institute for Pharmaceutical Sciences, Utrecht University 8125, Utrecht, the Netherlands; Universiteit Gent, Merelbeke, Belgium

**Keywords:** influenza, receptor, MHCII, neuraminidase, sialic acid

## Abstract

Influenza A viruses (IAVs) have a remarkable ability to adapt to new host species, which has been attributed to the specificity of the viral hemagglutinin (HA) for sialylated glycans and the functional balance between HA and the viral neuraminidase (NA). For decades, sialic acids on *N*- and *O*-linked glycans and glycosphingolipids were considered the only receptors that govern IAV entry, tropism, and species specificity. However, the discovery of bat-derived H17N10 and H18N11 viruses, followed by the identification of an avian H19 subtype, has fundamentally challenged this concept. These viruses use major histocompatibility complex class II (MHC-II) molecules as entry receptors independent of sialic acid binding. Moreover, recent studies demonstrate that certain H2N2 and H3N2 viruses exhibit dual receptor specificity, engaging both sialic acids and MHC-II, thereby expanding the conceptual understanding of IAV receptor usage. In this review, we discuss the evolving landscape of IAV receptor specificity by integrating classical insights on glycan-dependent attachment with emerging data on MHC-II-mediated entry. We discuss the structural determinants of sialic acid and MHC-II receptor engagement, the plasticity of HA receptor-binding sites, and the consequences of exclusive or dual receptor usage on host range and zoonotic potential. We further examine how MHC-II tropism shifts cellular targeting toward antigen-presenting cells and intestinal immune niches, and how this shift may relax selective constraints on NA function.

## INTRODUCTION

Wild waterfowl and shorebirds constitute the primary reservoir for influenza A viruses (IAVs), sustaining extensive genetic and antigenic diversity across viral subtypes that are defined by their surface glycoproteins: hemagglutinin (HA) and neuraminidase (NA). In their natural avian reservoir, IAVs replicate primarily in intestinal epithelia, typically causing subclinical infections and excreted by feces into the environment (1). Avian IAVs have spilled over into a broad range of species and established a remarkably complex ecology spanning diverse avian and mammalian hosts (2). In humans, four animal-derived IAVs caused worldwide pandemics since the beginning of the 20th century: the 1918 Spanish influenza (H1N1), 1957 Asian influenza (H2N2), 1968 Hong Kong influenza (H3N2), and 2009 Swine influenza (H1N1) (3). Descendants of these pandemic viruses have been maintained in human populations as seasonal IAVs causing annual waves of respiratory disease. A particularly unexpected expansion of the IAV host range was the discovery of genetically distinct IAV genomes in fruit bats from Central and South America (4–7). These New World bat IAVs diverge substantially from classical avian and mammalian IAVs and have provided new insights into the diversity of IAVs, both in sequence and in key biological properties (4, 5, 8–13). A major conceptual innovation from the discovery of bat IAVs was the identification of a previously unknown receptor usage. While the vast majority of IAVs rely on different structures of sialic acids for cell entry, a growing number of IAVs have now been shown to exploit a protein receptor to access their host cells: major histocompatibility complex class II (MHC-II).

In this review, we discuss the evolving landscape of IAV receptor specificity, with a particular focus on the paradigm-shifting discovery of MHC-II as a protein receptor, and explore its implications for host adaptation, tissue tropism, and zoonotic potential.

## DISCOVERY OF SIALIC ACID RECEPTORS FOR IAV HAs

**Fig 1:**
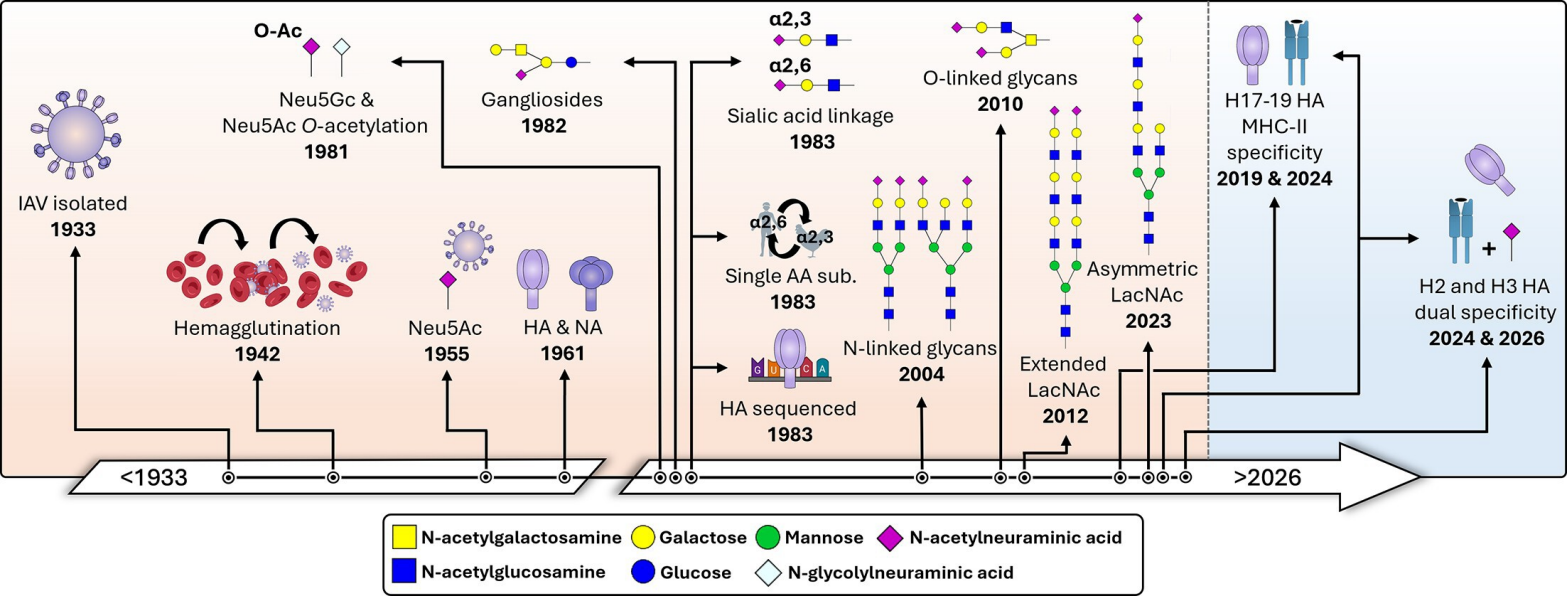
Timeline of major discoveries in influenza A virus (IAV) hemagglutinin (HA) receptor specificity. From left to right, the timeline illustrates key milestones in the understanding of IAV receptor usage: the initial discovery of IAVs in 1933; hemagglutination and receptor-destroying activity (1942); the identification of Neu5Ac as the classical cellular receptor (1955); separation of HA-binding and NA-cleavage functions (1961); recognition of other sialoforms, Neu5Gc and *O*-acetylated Neu5Ac, as additional receptors (1981); its capacity to bind ganglioside scaffolds (1982); differentiation between α2,3- and α2,6-linked sialic acid receptors (1983), alongside HA sequencing (1983) and demonstration that a single amino acid substitution can shift receptor specificity (1983); binding of sialylated N-glycan scaffolds (2004) and sialylated O-glycan scaffolds (2010); human H3N2 and H1N1 IAVs exhibit a preference for extended LacNAc-containing glycans (2012), whereas this binding was demonstrated to primarily require asymmetric branch extension (2023); MHC-II molecules identified as alternative protein entry receptors for HA subtypes H17-18 (2019) and H19 (2024); and recent evidence of dual receptor specificity in H2 (2024) and H3 (2026), engaging both sialic acids and MHC-II.

In 1942, Hirst and colleagues first demonstrated that viral preparations as well as the IAV HA alone were able to agglutinate chicken erythrocytes (Fig. 1) (14). Importantly, this agglutination was reversible upon prolonged incubation, rendering released erythrocytes incapable of rebinding and suggesting enzymatic cleavage of the, at the time, unidentified cellular receptor. Later, in 1955, Klenk and colleagues identified these cellular receptors as sialic acids by isolating them from urinary mucins following treatment with IAVs (15). Nevertheless, it was not until 1961 that it was shown that sialic acid binding and release are mediated by the action of two distinct viral surface glycoproteins, HA and NA, embedded into the IAV membrane (16).

**Fig 2:**
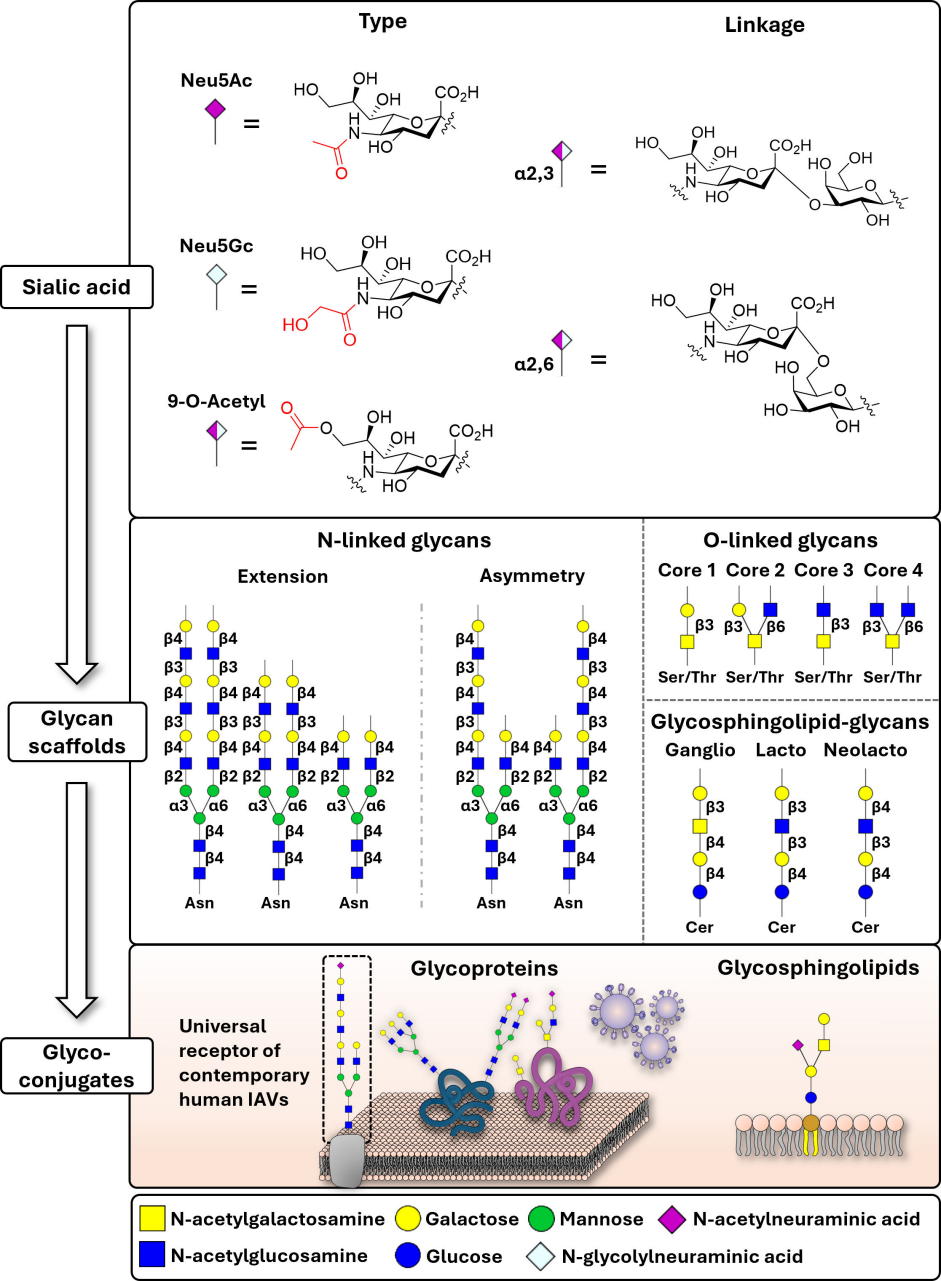
Structural features of glycan receptors known to influence recognition by IAVs, from sialic acid to glycoconjugate. From top to bottom: diverse sialic acid types and linkages, including O-acetylation of these terminal residues; glycan scaffolds comprising extended and asymmetric N-linked glycans, O-linked glycan cores, and glycosphingolipid glycan series; and their presentation on either glycoproteins or as glycosphingolipids. While N- and O-linked glycans are covalently attached to protein backbones, glycosphingolipids possess glycan moieties linked to ceramide lipids and are directly embedded in the plasma membrane. The indicated asymmetric N-linked glycan represents a receptor subset universally engaged by contemporary human IAVs.

It is now well established that the presence of these sialic acids, together with the sialic acid-binding specificity of a given HA, is the major determinant of IAV host range and tissue tropism (17). Sialic acids comprise a family of phylogenetically related α-keto acid monosaccharides that decorate glycoproteins and glycolipids and occur in a wide range of structural variants, collectively referred to as sialoforms (Fig. 2) (18). In humans, the predominant form is *N*-acetylneuraminic acid (Neu5Ac), also referred to as 5-amino-3,5-dideoxy-D-glycero-D-galacto-2-nonulosonic acid, whereas *N*-glycolylneuraminic acid (Neu5Gc) and *N*-hydroxylated 2-keto-3-deoxynonic acid (KDN) are additionally present in the epithelia of equine, bovine, and porcine host species (18–22). With the exception of KDN, both Neu5Ac and Neu5Gc can function as cellular receptors for HA (23). Notably, Neu5Gc is abundantly expressed in the equine respiratory tract and served as the major receptor for extinct equine H7N7 IAVs (20, 24), whereas contemporary circulating equine H3N8 IAVs, preferentially bind Neu5Ac. Conversely, avian H7N9 IAVs lost the ability to bind Neu5Gc after transmission from wild waterfowl to chickens, which solely express Neu5Ac (25). Recent data further suggest that adaptation of bovine-derived H5N1 IAVs may involve enhanced binding to Neu5Gc (26). Together, these findings emphasize the complex relationship between host adaptation and sialic acid specificity.

Depending on the host species, tissue, and cell type, sialic acids can also be modified with *O*-acetyl groups at various positions (Fig. 2). Whereas IAV HAs rarely tolerate *O*-acetylation of their cellular receptors, the hemagglutinin-esterase fusion protein (HEF) of influenza C and D preferentially binds and cleaves 9-*O*-acetylated Neu5Ac (27, 28). Beyond the terminal sialic acid itself, accumulating evidence indicates that the underlying glycan scaffolds dictate the HA:receptor interactions and thereby influence the viral tropism (29–32). In 1983, Rogers and colleagues first showed that the glycosidic linkage of Neu5Ac to the underlying glycan constitutes an essential host determinant, with avian IAVs preferentially binding α2,3-linked Neu5Ac and human-adapted IAV strains favor α2,6-linked Neu5Ac (33). Within the same year, Naeve and Webster sequenced the first HA gene (34), and Rogers and colleagues demonstrated that a single amino acid substitution in HA shifts the binding specificity from α2,3-linked to α2,6-linked Neu5Ac (33).

## GLYCAN SCAFFOLDS IN IAV HA BINDING

Sialic acids are present in distinct molecular contexts, including protein-linked *N*- and *O*-linked glycans as well as glycosphingolipids embedded directly into the cell membrane (Fig. 2). These scaffolds vary extensively in branch number, linkage configuration, chain length, and chemical modifications, thereby generating a high degree of structural complexity that modulates IAV-host interactions (18, 19).

Early studies by Chu and colleagues initially suggested that sialylated N-linked glycans were essential for productive IAV infections (35). In this study, Chinese hamster ovary (CHO) cells lacking functional N-acetylglucosaminyltransferase I (*GnT1*) and therefore deficient in complex N-glycan synthesis supported attachment but not infection with H1 and H3 IAVs. However, subsequent experiments using non-mutant CHO cells demonstrated that while sialylated N-glycans are important HA receptors, they are not essential for viral entry as other glycan scaffolds can also serve as functional receptors (36). Nevertheless, N-linked glycans remain the most extensively characterized subclass of glycan scaffolds in IAV infection (Fig. 2). Complex-type *N*-glycans are classified according to the number and spatial arrangement of branches extending from the α3- and α6-linked mannose residues. Asymmetry arises from variably elongated *N*-acetyllactosamine (LacNAc) motifs that can be further modified by fucosylation and sulfation, thereby generating a substantial structural diversity (18). Over the past two decades, comprehensive glycomic analyses have defined the N-glycan repertoires in human, porcine, and avian respiratory tract tissues (37–45). More recently, comparable analyses have been extended to avian intestinal tract tissues, the primary replication site of IAVs in their natural reservoir (45, 46).

Mucin-type *O*-linked glycans are attached to serine and threonine residues of proteins via an N-acetylgalactosamine (GalNAc) core. In contrast to N-glycans, O-glycans have been relatively underrepresented in experimental studies of IAV attachment, despite accumulating evidence supporting their contribution to IAV infections (47, 48). Among the eight known O-glycan core structures, cores 1 to 4 are most commonly expressed in mammalian and avian tissues (18), with core 1 and core 2 structures particularly abundant in the respiratory tract and therefore likely representing physiologically relevant IAV HA receptors (Fig. 2) (38–41, 45). Direct evidence for HA:O-glycan interaction was first provided by studies showing that various H1N1 IAVs bind protein-linked O-glycans (49).

Multiple studies have demonstrated that glycosphingolipids promote attachment and entry through distinct yet interrelated molecular mechanisms (50–56). More recent experiments using CRISPR-engineered cells displaying defined glycan repertoires further corroborated the role of glycosphingolipids as entry receptors for IAVs (51, 57). Glycosphingolipids are membrane-embedded glycolipids that consist of a galactose (Gal) or glucose (Glc) linked to a ceramide backbone, with sialylated members collectively termed gangliosides (Fig. 2) (18, 54, 55). Despite their widespread presence, detailed characterization of their tissue- and species-specific composition remains largely limited to human and porcine lung tissues (38, 43). In humans, ganglio-, lacto-, and neolacto-series of gangliosides are most abundant and likely constitute physiologically relevant HA receptors. The capacity for IAV HAs to bind gangliosides was first demonstrated in 1982 using neuraminidase-treated erythrocytes reconstituted with defined, exogenously incorporated ganglioside structures (55). These experiments revealed that HA binding depends not only on the presence of sialic acids, but also on the specific ganglioside architecture.

## ADAPTATIONS IN RECEPTOR-BINDING PREFERENCES OF IAV HAs

The receptor-binding specificity of IAV HAs continuously evolves to optimize viral fitness within distinct host environments, thereby influencing viral infection efficiency, transmission, tropism, and zoonotic potential. Adaptation of HA-binding specificity can also affect viral replication properties in traditional systems, such as embryonated chicken eggs. This became particularly evident for human H3N2 IAVs, whose evolving receptor preferences no longer matched the glycan repertoire of egg-based propagation systems, ultimately contributing to the exclusion of A/Fujian/411/2002 from the 2003–2004 influenza vaccine formulation. The older A/Moscow/10/99-like virus was instead adopted as the H3N2 vaccine component, resulting in a reduced antigenic match with circulating H3N2 IAVs and spurring research into replacing egg-based vaccine production (58).

A major shift in receptor-binding preferences of human-adapted IAVs became apparent with the development of glycan microarray technologies. In 2012, a glycan microarray screening of a large panel of bi-antennary N-glycans demonstrated that certain human IAV HAs preferentially bind to receptors containing multiple consecutive LacNAc repeats (59). Subsequent studies examining the HA-binding specificity of human H3N2 IAVs isolated between 1968 and 2012 revealed a progressive narrowing of receptor specificity toward N-glycans with di- and tri-LacNAc repeats (60, 61). After 1989, many H3N2 IAVs exhibited highly selective binding to glycans harboring at least three LacNAc repeats (62–64). A similar trend was observed for human-adapted H1N1 IAVs, with the 2009 pandemic strain A/California/04/2009 preferentially binding N-glycans with three or more consecutive LacNAc repeats (64, 65), indicating human-adapted IAVs increasingly depend on highly complex glycan structures for efficient receptor engagement. Conclusively, during evolution in humans, IAVs gradually lost the ability to bind mono-LacNAc structures.

In addition to the symmetric extension of N-glycan LacNAc branches, asymmetric extensions can also influence IAV HA-binding properties (62). Glycan microarray screening using asymmetric N-glycans demonstrated that modern post-pandemic human H1N1 IAVs require at least one sialylated di-LacNAc branch for efficient binding (65). Both human-adapted H1N1 and H3N2 IAVs frequently exhibit a preference for glycan extension and terminal sialylation on the α3-branch bi-antennary N-glycans (63), highlighting that branch topology contributes substantially to receptor specificity. This subset of asymmetric N-glycans is now widely accepted as the universal receptor for contemporary human IAVs in the human respiratory tract.

Recent findings indicate that adaptations in IAV HA specificity extend beyond the classical avian-to-human α2,3-to-α2,6 sialic acid receptor paradigm and involve increasingly complex receptor interactions with distinct glycan scaffolds. Human isolates of avian-origin H7N9 IAVs acquired the Q226L substitution (H3 numbering), which is generally associated with a switch in avian-to-human receptor preference. Surprisingly, however, this substitution did not substantially enhance binding of conventional α2,6-linked Neu5Ac-containing glycan receptors. Instead, the adapted H7 HA gained the ability to recognize O-glycans with α2,3-linked Neu5Ac attached to both branches (66). Recently, Weber and colleagues demonstrated that contemporary clade 2.3.4.4b H5N1 viruses exhibit a distinct specificity for core 1 and core 3 O-glycans containing α2,3-linked Neu5Ac, a property that may contribute to their expanded host range and unusual tissue tropism (67).

## NA FUNCTIONALITIES

Almost two decades after the discovery of sialic acid-dependent hemagglutination, the receptor-destroying enzyme responsible for particle release, the viral NA, was isolated as an active preparation from IAVs (Fig. 1) (16). NA activity is particularly important during virion budding, preventing self-aggregation and promoting efficient particle release. Additionally, NA has been implicated in early stages of infection, including facilitation of viral movement through sialylated mucus barriers and modulation of HA-mediated attachment events (68–70). A central dogma in IAV research is the requirement for a functional balance of HA binding and NA cleavage (71–74). The functional balance between HA binding and NA cleavage has been shown in several studies, in which experimental perturbation of this balance results in compensatory adaptations: reduced NA activity selects for decreased HA-binding affinity, and diminished HA-binding affinity permits lower NA activity (75). However, the discovery of the bat-derived N10, N11, and N12 NAs, which lack sialidase activity, challenged the universality of this paradigm. Biochemically, classical IAV NA does have a remarkable substrate promiscuity towards both Neu5Ac and Neu5Gc, with a clear preference for Neu5Ac (20, 76, 77), consistent with the generally poor binding of HA to Neu5Gc. In contrast, O-acetylated sialic acids are inefficiently cleaved by NA (78–80); indeed, 4-O-acetylated sialic acids, which are enriched in horse and guinea pig serum glycoproteins but absent in humans, can function as competitive inhibitors of IAVs (81). Regarding linkage specificity, human-adapted N1 and N2 NAs have progressively gained the ability to cleave α2,6-linked Neu5Ac while retaining the capacity to cleave α2,3-linked Neu5Ac. This broadened substrate specificity of NA contrasts with the binding specificity of HA, which typically display an exclusive specificity for α2,6-linked Neu5Ac binding during human host adaptation (82). Nevertheless, despite glycan microarray analyses of NA specificity (73, 83), the detailed substrate specificity remains incompletely understood, partly due to methodological limitations such as the lectin-based detection of exposed galactose residues, which imposes intrinsic specificity biases.

## DISCOVERY OF MHC-II-DEPENDENT IAV HAs

**Fig 3:**
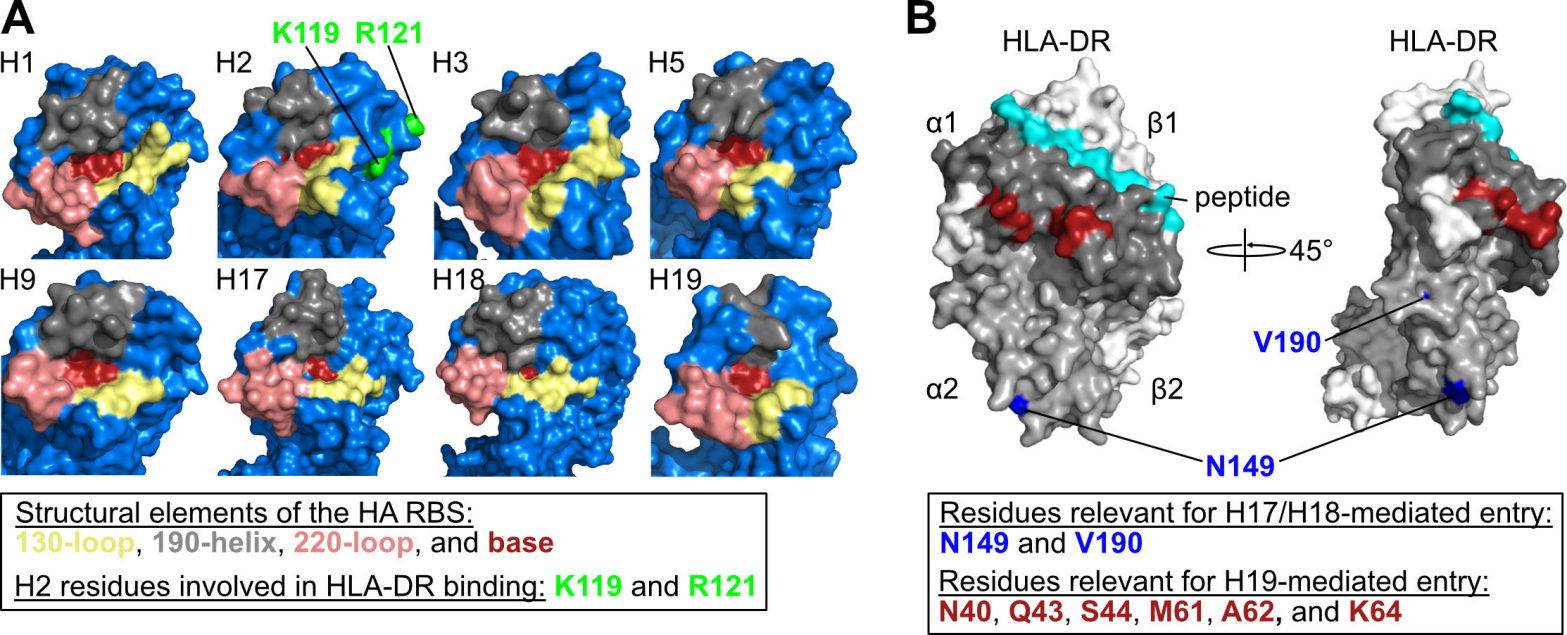
Structural domains within different IAV HA head domains and human HLA-DR. (**A**) The RBS within monomeric head domains of different IAV HA proteins, comprising the 130-loop (yellow), 190-helix (gray), and 220-loop (rose) regions, as well as the base (dark red), is highlighted. Structural models are shown for the HA proteins: H1 of A/California/04/2009(H1N1) (pdb: 4M4Y), H2 of A/Japan/305-/1957(H2N2) (pdb: 3KU3), H3 of A/Singapore/H2011.447/2011(H3N2) (pdb: 4WE8), H5 of A/Vietnam/1194/04(H5N1) (pdb: 2IBX), H9 of A/swine/Hong Kong/9/98(H9N2) (pdb: 1JSD), H17 of A/little yellow-shouldered bat/Guatemala/060/2010(H17N10) (pdb: 4H32), H18 of A/flat-faced bat/Peru/033/2010(H18N11) (pdb: 4K3X), and H19 of A/lesser scaup/California/3087/2010(H19Nx) (pdb: 8VCC). Note that residues K119 and R121 in H2, which are involved in HLA-DR binding, are highlighted in green. Residue S107 is located on the backside and is not indicated. (**B**) Structural representation of the peptide-loaded human MHC-II molecule HLA-DR (pdb: 1DLH). Residues N149 and V190, which have been shown relevant for H17/H18-mediated entry, are highlighted in blue, whereas residues N40, Q43, S44, M61, A62, and K64, required for H19-mediated entry, are shown in dark red.

The long-standing dogma that sialic acids serve as the exclusive receptors for all IAVs was challenged by the discovery of previously unknown IAV sequences in rectal swab material from the little yellow-shouldered bat (*Sturnira lilium*) in Guatemala and the flat-faced fruit bat (*Artibeus planirostris*) in Peru by Tong and colleagues (4, 5). Early sequence analysis revealed that, with the notable exception of the HA sequences, the genomes of these bat-derived viruses (H17N10 and H18N11) are only distantly related to those of classical IAVs (4, 5, 11). Consistent with this, they are unable to reassort with the latter, due to molecular incompatibilities, both on protein and RNA level (8, 9). Importantly, all biochemical experiments with purified H17/H18 HA and N10/N11 NA proteins failed to detect the well-established sialic acid-binding and neuraminidase activity, respectively. Structural analysis demonstrated that the head domains of H17 and H18 HA retain the canonical 130-loop, 190-helix, and 220-loop that together form the receptor binding site (RBS) of classical HAs (84) (Fig. 3A). However, multiple amino acid substitutions result in a flattened and widened RBS that is incompatible with sialic acid binding (5, 85). Although the head domains of N10 and N11 display overall tertiary structural similarity to classical NAs, critical and highly conserved residues in the catalytic domain are substituted, leading to a widened active site (5, 86), similar to the H17 and H18 HA RBS. Collectively, these findings suggested early on that sialic acids were not the entry receptors for the newly discovered bat-derived IAVs.

Initial attempts to propagate H17N10 and H18N11 viruses in primary bat tissue cultures failed, and efforts to generate infectious bat IAVs by reverse genetic approaches were not successful (8, 9, 87). Nevertheless, viral particles were detected by electron microscopy in the supernatant of producer cells used for virus rescue, indicating that viral assembly and release occurred, but that the commonly used cell lines for IAV propagation lacked the appropriate entry receptor. To identify cell lines susceptible to H17 and/or H18-mediated entry, recombinant vesicular stomatitis virus (VSV) particles were generated in which the VSV glycoprotein was replaced by either H17 (VSV-H17) or H18 (VSV-H18). Using these pseudotyped viruses, several human, canine, and bat cell lines were identified as susceptible to H17- and H18-mediated entry (88). Subsequent genome-wide CRISPR-Cas9 knockout screens, together with transcriptomic comparisons of susceptible and non-susceptible cell lines, identified major histocompatibility complex class II (MHC-II) molecules as the entry receptor for both H17 and H18 (10) (Fig. 1). Consistently, genetic ablation of MHC-II rendered susceptible cells non-permissive to infection with VSV-H17 and VSV-H18, and authentic H18N11 failed to replicate in MHC-II-deficient mice, whereas replication was observed in wild-type animals. As the CRISPR-Cas9 screens did not reveal any additional receptor candidates, these findings suggest that MHC-II serves as the sole cellular entry receptor for the bat-derived IAVs H17N10 and H18N11. Consistent with this, entry of bat-derived IAVs was efficiently inhibited by the addition of soluble MHC-II complexes in a dose-dependent manner (89).

Beyond the human MHC-II molecules (HLA-DR, HLA-DQ, and HLA-DP), H18 and, to a lesser extent, H17 were shown to engage MHC-II molecules from a wide range of species, including multiple bat species as well as pigs, mice, and chickens, highlighting a pronounced cross-species compatibility at the receptor level (Table 1) (10). This broad compatibility is notable because MHC-II molecules are conserved throughout vertebrate evolution but differ substantially between species and alleles. In humans, classical MHC-II molecules are encoded by the HLA-DR, HLA-DQ, and HLA-DP loci. Other vertebrates possess species-specific orthologous systems. For example, pigs encode SLA-DR and SLA-DQ, which correspond to human HLA-DR and HLA-DQ, and mice encode H-2A and H-2E, which broadly correspond to human HLA-DR and HLA-DQ, respectively (90–92). In contrast, avian species possess a distinct MHC organization, exemplified by the compact chicken B locus (92, 93). Despite this nomenclatural and genetic diversity, all MHC-II molecules share a common heterodimeric architecture consisting of an α (α1 and α2 domains) and a β chain (β1 and β2 domains). While the α1 and β1 domains form the peptide-binding groove, the α2 and β2 domains provide the structural basis (92, 94).

**TABLE 1:** Summary of currently known IAV strains with MHC-II specificity

Origin	Isolate	Subtype	MHC-II specificity
Human	A/JP/305/1957	H2N2	*Homo sapiens* HLA-DR (95); *Sus scrofa* SLA-DR (95); *Mus musculus* H-2E (95); *Gallus gallus* B-L (95); *Anas platyrhynchos Ap*-DR (95); *Cygnus atratus Ca*-DR (95)
	A/Victoria/361/2011	H3N2	*Homo sapiens* HLA-DR (96); *Sus scrofa* SLA-DR (96)
Swine	A/turkey/Ohio/313053/2004	H3N2	*Homo sapiens* HLA-DR (96); *Sus scrofa* SLA-DR (96)
New World leaf-nosed bats (Phyllostomidae)	A/little yellow-shouldered bat/Guatemala/060/2010	H17N10	*Homo sapiens* HLA-DR (13), HLA-DQ, HLA-DP (10); *Sus scrofa* SLA-DR (10); *Eptesicus fuscus Ef*-DR (10; *Myotis lucifugus Ml*-DR (10)
	A/little yellow-shouldered bat/Guatemala/164/2009		
	A/little yellow-shouldered bat/Guatemala/153/2009		
	A/flat-faced bat/Peru/033/2010	H18N11	*Homo sapiens* HLA-DR, HLA-DQ, HLA-DP (10); *Sus scrofa* SLA-DR (10); *Mus musculus* H-2A, H-2E (10); *Eptesicus fuscus Ef*-DR (10); *Myotis lucifugus Ml*-DR (10); *Pteropus alecto Pa*-DR (10); *Artibeus jamaicensis Aj-*DR (97); *Gallus gallus* B-L (10); *Anas platyrhynchos Ap*-DR (95); *Cygnus atratus Ca*-DR (95); *Aythya fuligula Af*-DR (98)
	A/dark fruit-eating bat/Bolivia/PBV780-781/2011		
	A/Artibeus lituratus/Brazil/2344/2012		
	A/Artibeus lituratus/Brazil/2301/2012		
Fishing bats (Noctilionidae)	A/fishing bat/Colombia/2023	H18N12	
North American duck (Anatidae)	A/lesser scaup/California/3087/2010	H19Nx	*Pteropus alecto Pa*-DR (98); *Mus musculus* H-2E (98); *Aythya fuligula Af*-DR (98)
	A/lesser scaup/California/1742/2013		

Although a direct structural interaction between H17/H18 and MHC-II has not yet been experimentally resolved, *in silico* modeling of the H18:HLA-DR interaction identified the α2 domain as the principal interface mediating HA binding (97). Consistent with this model, targeted mutagenesis demonstrated that the highly conserved residues N149 within loop 4 and V190 within β-sheet 6 of the α2 domain are essential for H17- and H18-mediated entry (Fig. 3B). Despite these advances, the exact receptor-binding domain within H17 and H18 remains unknown.

Recent data indicate that the use of MHC-II for cell entry is not restricted to the bat-derived IAV subtypes H17N10 and H18N11 (Table 1). In 2023, Fereidouni and colleagues identified a genetically distinct IAV HA sequence isolated from a cloacal swab of a common pochard (*Aythya ferina*) in Kazakhstan (99). Owing to its distant relationship to known H9 sequences, the HA was classified as a novel subtype, H19. Closely related H19 sequences have since been detected in H19Nx IAVs isolated from lesser scaup ducks (*Aythya affinis*) in California, USA (98). Functional analyses of receptor specificity by Karakus and colleagues demonstrated that H19 lacks detectable sialic acid-binding activity and mediates cell entry in an MHC-II-dependent manner. In contrast to H18, which can engage a broad range of MHC-II molecules across all tested species, H19 exhibits a more restricted specificity, as it so far was only shown to be compatible with MHC-II molecules derived from waterfowl and mice (Table 1). Structurally, the head domain of H19 contains a conserved RBS formed by the canonical 130-loop, 190-helix, and the 220-loop (Fig. 3A). Notably, of the amino acid residues critical for sialic acid binding, only two positions differ from classical IAV HAs: G134T and G/Q226I (H3 numbering). This limited divergence contrasts sharply with H17 and H18, which harbor extensive amino acid substitutions in the head domain. Consequently, targeted mutagenesis of H19 to introduce the substitutions T134G, I226Q, and P137A within the 130-loop of the RBS was sufficient to restore sialic acid binding (98), underscoring the structural plasticity of the H19 RBS and its evolutionary proximity to classical sialic acid-binding HAs. Yet, the MHC-II binding site and the amino acids required for MHC-II-dependent entry in H19 remain currently unknown. As for H17 and H18, direct biochemical evidence for an interaction between H19 and MHC-II molecules is still lacking. However, mutagenesis studies have demonstrated that the MHC-II α1-chain is critical for H19-mediated cellular entry. Specifically, amino acid residues 40, 43, 44, 61, 62, and 64, located near the peptide-binding groove, were identified as essential determinants (10) (Fig. 3B). Although the MHC-II domain required for H17- and H18-mediated entry differs from those involved in H19 entry, it is notable that across all MHC-II-dependent HAs characterized to date, receptor engagement appears to rely predominantly on the MHC-II α-chain (97, 98). The mechanistic basis for this preference remains unclear; however, compared with the α-chain, the β-chain exhibits greater polymorphism (100).

## HAs WITH DUAL RECEPTOR SPECIFICITY

In addition to using either sialic acids or MHC-II molecules for host cell entry, some IAVs have HAs with dual-receptor specificity, enabling entry via both receptor classes. Studies of different H2N2 viruses revealed that several Eurasian avian strains, as well as human isolates, including the 1957 pandemic virus, exhibit such dual receptor specificity (Fig. 1) (95). Interestingly, MHC-II molecules derived from humans, pigs, and waterfowl can serve as alternative entry receptors for these viruses and are engaged by H2s independently of the classical, sialic acid-specific RBS (Table 1) (95). Instead, H2s with a dual receptor specificity harbor a conserved sequence motif at the outer edge of the head domain, comprising the residues S107, K119, and R121 (H3 numbering), that allows for MHC-II-dependent cell entry (Fig. 3A). The avian origin of the H2 of the 1957 pandemic virus is of particular interest, as for other avian H2 IAVs there is a positive correlation between the presence of this MHC-II entry motif and a marked zoonotic risk scoring based on their ability to replicate in primary human airway cells and in ferrets, despite the lack of human-like α2,6-sialic acid receptor specificity (12, 101). More recently, it became evident that H2N2 viruses are not the only IAVs with dual receptor specificity. Using a pair of H3N2 viruses, Cardenas and colleagues found that a prototypic human-seasonal and a swine-adapted H3N2 virus engage MHC-II as an alternative entry receptor in a species-specific manner (Fig. 1) (96). While the human MHC-II molecule HLA-DR, but not the porcine SLA-DR enabled efficient entry of the human H3N2 in desialylated cells, the porcine H3N2 was able to engage SLA-DR and to a certain degree also HLA-DR as a receptor (Table 1). Interestingly, experimental infection of pigs with a human-seasonal H3N2 virus resulted in the emergence of individual HA substitutions at residues A138S, V186G, or F193Y (H3 numbering), which are located in proximity to the RBS (102). These substitutions altered sialic acid binding preferences and increased specificity for the porcine SLA-DR (96), suggesting that structural changes at the sialic acid-specific RBS can modulate recognition of both sialylated glycans and MHC-II. These results thus suggest that dual receptor specificity may facilitate cross-species transmission and may have contributed to pandemic emergence in the past (103). From an evolutionary perspective, the discovery of H2s and H3s with dual receptor specificity in avian and porcine species offers a new model for understanding the emergence of the exclusive MHC-II dependence observed in H17, H18, and H19. Rather than emerging through multiple independent evolutionary events, MHC-II usage may have evolved from an ancestral HA with dual-receptor specificity that lost its sialic acid binding capacity due to host-specific adaptations, such as changes in cell tropism. However, the three residues identified for H2 to mediate engagement of MHC-II are absent or are only partially conserved among H17-H19. This might indicate that these viruses have evolved to interact with MHC-II in a different manner or, alternatively, there are additional, conserved MHC-II binding residues in HA, which are yet to be identified.

## ANOTHER RECEPTOR, ANOTHER TROPISM?

MHC-II molecules are primarily expressed on professional antigen-presenting cells (APCs), including dendritic cells, macrophages, and B cells. These cells internalize extracellular pathogens, process their proteins, and present antigenic peptides via MHC-II molecules to CD4^+^ T cells, thereby initiating adaptive immune responses (104, 105). In addition, MHC-II molecules are expressed in the thymus, where they play a central role in T cell selection (106). Constitutive MHC-II expression is also observed in certain non-hematopoietic cell types, most notably in intestinal epithelial cells of the small intestine (107). Under inflammatory conditions, MHC-II expression can further be induced in additional cell types, including T cells (108). This comparatively restricted and cell-type-specific expression pattern of MHC-II contrasts sharply with the near-ubiquitous distribution of sialic acids across different cell types and tissues. Consequently, the tissue tropism of MHC-II-dependent IAVs is not governed by broadly displayed glycan receptors, but rather by the availability of specialized immunological cell populations and tissues.

To date, the tropism of MHC-II-dependent IAVs has been systematically investigated only for H18N11 (109, 110). Following oronasal inoculation, H18N11 replicated in the olfactory epithelium of mice, and additionally infected the pharyngeal and palatine tonsils in ferrets. In the natural host, the Jamaican fruit bat (*Artibeus jamaicensis*), oronasal inoculation resulted in infection of the squamous epithelium of the palatine tonsils, the lamina propria of the small intestine, and the follicle-associated epithelium of the jejunal Peyer’s patches (109). Integration of single-cell RNA sequencing with spatially resolved laser scanning microscopy further demonstrated that H18N11 infects and replicates in both Jamaican fruit bat intestinal epithelial cells and intestinal leukocytes, particularly macrophages (110). This cellular tropism was corroborated by productive infection of human PBMC-derived macrophages and Jamaican fruit bat bone marrow-derived macrophages with H18N11. Overall, these findings indicate that the *in vivo* tropism of H18N11 closely reflects the expression pattern of MHC-II within the intestinal epithelium and immune compartments. However, the evolutionary basis of leukocyte infection, as opposed to the epithelial tropism for classical IAVs, and the functional contribution of infected leukocytes to the viral life cycle remain unresolved. Other viruses, such as HIV-1 (111), measles virus (112, 113), and Epstein-Barr virus (114), infect immune cells not only as sites of replication, but also as vectors for intra-host dissemination, immune modulation, and the establishment of systemic or persistent infection. In this context, infection of MHC-II-expressing leukocytes by H18N11 may facilitate viral spread within the intestinal immune network and promote dissemination via the lymphatics. Moreover, targeting immune cells may enable modulation of local innate and adaptive immune responses, thereby shaping a tissue environment permissive for viral replication. It remains currently unknown whether MHC-II-dependent IAVs, such as H18N11, primarily infect leukocytes for viral dissemination, immune modulation, or both, but uncovering the precise infection dynamics might be central to understanding the biology and evolutionary advantages of this cellular tropism.

Although studies on the tropism of the duck-derived H19Nx virus are currently lacking, the isolation of the viral genomes from rectal samples (98, 99) strongly suggests that replication occurs primarily in the gastrointestinal tract, as for to other low-pathogenic avian influenza viruses (LPAIVs) in waterfowl (1). The MHC-II dependency of the duck-derived H19 thus raises fundamental and evolutionary questions when compared with the remaining classical sialic acid-dependent H1-H16 subtypes circulating in wild waterfowl. A comparative analysis of classical IAVs and MHC-II-dependent H19Nx viruses in their natural reservoirs would therefore provide an ideal mechanistic framework to dissect how HA-mediated receptor engagement determines viral entry routes, cell-type specificity, and infection dynamics within the intestinal environment. Such studies could further clarify whether engagement of MHC-II molecules as entry receptors represents an evolutionary adaptation to the mucosal immune niche, promoting efficient viral replication while concurrently shaping local immune responses. In this context, as the precise cell types infected in the gastrointestinal tract of avian species has not yet been resolved, it is tempting to speculate that dual receptor specificity, as observed for certain H2s and H3s, has expanded the cellular tropism for IAVs in the gastrointestinal tract of their natural waterfowl reservoirs. This hypothesis is supported by infection studies in mice, showing that viruses carrying an H2 with dual receptor specificity infect a significantly higher number of MHC-II-positive dendritic cells (CD11b^+^/CD11c^+^ population) in the lungs compared with viruses with an exclusively sialic acid-dependent H1 HA (95). Because MHC-II-binding properties apparently originate from avian-adapted H2 IAVs, future studies should test whether dual receptor specificity confers a fitness advantage in avian hosts by expanding the cellular tropism compared with exclusively sialic acid-dependent variants. It is also noteworthy that this dual receptor specificity originating from the avian reservoir did not appear to undergo negative selection after transmission to humans. Sequence analyses of available human-derived H2-HA sequences between 1957 and 1968 show that the MHC-II entry motif in the HA head domain was preserved throughout circulation of the H2N2 virus in the human population. At the same time, earlier histopathological and immunohistochemical studies of H2N2-infected human lung samples predominantly showed infection of tracheobronchial and alveolar epithelial cells (115, 116). This suggests that, although H2N2 viruses can engage MHC-II in humans, this neither caused an apparent shift in cellular tropism nor resulted in a significant fitness advantage.

## THE FUNCTION OF NON-CLASSICAL NAs REMAINS UNCLEAR

Unlike H17 and H18 HAs, the function of unconventional N10 and N11 NAs, as well as the recently identified N12 NA, remains enigmatic. Functional characterization is complicated by the fact that, under *in vitro* conditions, neither N10 nor N11 NA is essential for viral replication or particle release (88, 109). Accordingly, serial passaging of wild-type H18N11 by Ciminski and colleagues on endogenously MHC-II-expressing MDCK II cells resulted in rapid counterselection of the full-length N11 NA, either through the acquisition of a premature stop codon or through large internal deletions of the viral RNA segment encoding N11 (109). Similarly, infection of mice with H18N11 led to the selection of viral variants carrying deletions within the N11 segment. Nevertheless, additional evidence points to context-dependent selection on N11 in non-bat hosts. Following experimental inoculation of ferrets with an N11-deficient H18N11 variant, but not the wild-type H18N11, replicated to detectable levels in organs of ferrets, yet viruses recovered from the lungs and nasal conchae contained variants with restored full-length N11. In parallel, Zhong and colleagues obtained H18N11 variants after *in vitro* passaging in MDCK II cells that harbored two amino acid substitutions in the N11 head domain (117). These variants exhibited increased replication properties in human cell lines, as well as in mice and ferrets. However, because sequencing of viruses isolated from the organs after experimental inoculation was not performed, it remains unclear whether the wild-type H18N11 and the N11 mutant variants were genetically stable after replication *in vivo*. Taken together, these findings suggest that selective pressure on N11 NA is host- and tissue-dependent and may differ substantially outside the natural bat reservoir. Compelling evidence for a functional relevance of N11 NA has emerged so far most convincingly in the context of infection of the natural bat host. In Jamaican fruit bats, wild-type H18N11 replicated efficiently and was horizontally transmitted to naive contact bats, whereas an N11-deficient H18N11 variant replicated but failed to transmit, suggesting that N11 NA contributes to dissemination across the intestinal mucosal epithelia or bat-to-bat transmission (109). Consistent with this idea, all currently known bat-derived IAVs, including H17N10, H18N11, and H18N12, retain a complete NA gene segment.

Among the MHC-II-dependent IAVs, the duck-derived H19Nx virus appears to be an exception, as both available isolates carried either no NA segment or only a truncated NA segment with a large deletion encompassing both the stalk and head domains (98). This pattern mirrors the rapid counterselection of the intact N11 segment observed during *in vitro* passaging of H18N11 (109). Notably, the 232-nucleotide NA fragment recovered from the H19Nx isolate shared 99% sequence identity with the classical N6 NA of A/duck/Minnesota/104/1974 (98), arguing for a closer relationship to a classical avian NA than to the unconventional, genetically divergent bat IAV N10 and N11 NAs. This unexpected genomic constellation raises several mechanistic questions: why did H19 HA not co-evolve with an N10/N11-like NA, as observed for H17 and H18 HA, but instead emerge in a virus with a fragmented, or absent NA segment? Furthermore, if lesser scaup ducks indeed represent the natural reservoir of H19Nx, how could HA-autonomous replication and transmission be sustained, given that efficient spread of classical IAVs relies on functional interdependence between HA-mediated receptor binding and NA-mediated receptor cleavage? One plausible explanation is that the shift in receptor usage from sialic acids to MHC-II fundamentally changes the selective constraints on NA. If virion attachment is no longer mediated by sialic acids, the canonical requirement for NA-driven receptor release may be diminished, potentially rendering NA activity dispensable under certain conditions. Finally, this finding indirectly implies that N10 and N11 NA do not possess classic receptor-destroying activity; otherwise, it would be difficult to explain why NA loss or deletions are repeatedly tolerated or selected in MHC-II-dependent viruses.

## SUMMARY

Since the 1940s, sialic acids on N- and O-linked glycans and glycosphingolipids have been recognized as the primary receptors for the majority of IAVs. The recent discovery of MHC-II molecules as entry receptors for H17, H18, and H19 subtypes challenged this paradigm and raised the possibility that classical IAV subtypes might also engage MHC-II. Indeed, certain H2N2 and H3N2 viruses were most recently shown to exhibit dual receptor specificity, using both sialic acids and MHC-II molecules for host cell entry. However, the evolutionary origins of this dual receptor specificity, the basis for exclusive MHC-II dependence in certain IAVs, and the selective pressures favoring MHC-II usage remain obscure. As MHC-II is predominantly expressed on the surface of APCs, targeted infection of these cells could confer an evolutionary advantage due to a broadened cellular tropism. Yet, both exclusive MHC-II usage and dual receptor specificity appear rare among IAVs, suggesting that these traits emerge only under specific circumstances. The NA proteins of the MHC-II-dependent subtypes H17N10, H18N11, and H18N12 are unconventional as they lack canonical sialidase activity. Although their precise function still remains unknown, infection studies with H18N11 showed that N11 is dispensable *in vitro*, but contributes to enhanced transmission in the natural bat host. Similarly, duck-derived H19Nx isolates replicate in the absence of a functional NA, indicating that exclusive MHC-II receptor usage fundamentally relaxes selective pressure on NA-driven sialic acid cleavage.
